# Combining Low-Light Scene Enhancement for Fast and Accurate Lane Detection

**DOI:** 10.3390/s23104917

**Published:** 2023-05-19

**Authors:** Changshuo Ke, Zhijie Xu, Jianqin Zhang, Dongmei Zhang

**Affiliations:** 1 School of Science, Beijing University of Civil Engineering and Architecture, Beijing 102616, China; 2102520021001@stu.bucea.edu.cn (C.K.); zhangdm927@163.com (D.Z.); 2 School of Geomatics and Urban Spatial Informatics, Beijing University of Civil Engineering and Architecture, Beijing 102616, China; zhangjianqin@bucea.edu.cn

**Keywords:** lane detection, low-light enhancement, semantic segmentation, image classification

## Abstract

Lane detection is a crucial task in the field of autonomous driving, as it enables vehicles to safely navigate on the road by interpreting the high-level semantics of traffic signs. Unfortunately, lane detection is a challenging problem due to factors such as low-light conditions, occlusions, and lane line blurring. These factors increase the perplexity and indeterminacy of the lane features, making them hard to distinguish and segment. To tackle these challenges, we propose a method called low-light enhancement fast lane detection (LLFLD) that integrates the automatic low-light scene enhancement network (ALLE) with the lane detection network to improve lane detection performance under low-light conditions. Specifically, we first utilize the ALLE network to enhance the input image’s brightness and contrast while reducing excessive noise and color distortion. Then, we introduce symmetric feature flipping module (SFFM) and channel fusion self-attention mechanism (CFSAT) to the model, which refine the low-level features and utilize more abundant global contextual information, respectively. Moreover, we devise a novel structural loss function that leverages the inherent prior geometric constraints of lanes to optimize the detection results. We evaluate our method on the CULane dataset, a public benchmark for lane detection in various lighting conditions. Our experiments show that our approach surpasses other state of the arts in both daytime and nighttime settings, especially in low-light scenarios.

## 1. Introduction

Lane detection is a critical and hot task for ensuring safe autonomous driving and advanced driver assistance system (ADAS) [1]. Computer vision technology has facilitated significant advancements in this field, making it a widely researched topic in academia and industry alike. The ability to accurately detect lanes plays a crucial role in guiding vehicles to travel safely on the road.

Various deep learning methods have emerged in lane detection in recent years, enhancing the detection performance and adaptability under diverse scenarios. Nevertheless, the task still faces several challenges that need to be overcome. One of the most pressing issues is the robustness of models to various adverse conditions in real-world scenarios, such as extreme light [2] and weather variations. Furthermore, lane markings are often occluded by other objects, such as cars, which frequently occurs in autonomous driving situations. This challenge requires lane detection models to possess high-level semantic understanding of the scene and be able to distinguish between lane markings and other objects accurately. Although the traditional method is simple and fast, the detection results are often not satisfactory. Addressing these challenges is crucial to enable the reliable deployment of autonomous driving systems in real-world environments. To more efficiently utilize visual information, there are specialized convolution operations that facilitate the aggregation of information from various dimensions in the segment model of SCNN [3]. These operations involve the processing of slice features, which are then added one by one to enable the information aggregation. Despite its effectiveness, this method is relatively slow to use in practice. SAD [4] has emerged as a promising approach to reduce the parameters required for lane detection. Despite its potential, SAD still suffers from suboptimal processing speed, which hinders its practical applicability. UFLD [5] transform this task into a simpler classification problem, which has the advantage of significantly reducing the computational overhead and enabling ultra-fast lane detection. However, the accuracy of UFLD still needs improvement, as it does not perform well in low light environments or for capturing small targets and details.

In this paper, we present a novel approach named low-light enhancement fast lane detection (LLFLD), which leverages the automatic low-light scene enhancement network (ALLE) network for image adaptive enhancement prior to detection. Specifically, we introduce a perceptual brightness threshold and apply enhancement detection to images that fall below this threshold, resulting in improved detection performance in low-light scenarios. To further increase accuracy, we leverage the symmetric distribution of lane markings in the driving perspective and design a symmetric feature flipping module (SFFM) that enhances low-level features for more precise lane localization. In addition to the main branch for detection, we present an auxiliary segmentation module that is only activated during training.

In our auxiliary segmentation module, we incorporate the channel fusion self-attention mechanism (CFSAT) to enhance the acquisition of global context information. This is achieved by establishing connections between the lane markings and the wider global feature map. Moreover, our approach involves the introduction of a novel structural loss function that is based on the geometric shapes of lanes. Through this loss function, we optimize the segmentation and classification performance of our model by leveraging the inherent structure of the lane markings. By employing these strategies, we are able to effectively capture important contextual features while simultaneously mitigating unnecessary redundancy, resulting in a more robust and efficient detection algorithm.

Our approach has been tested extensively using the benchmark CULane dataset [3], and we present comprehensive experimental results along with comparisons to other state-of-the-art methods. Additionally, we conducted an ablation study to analyze the impact of our design choices on the performance of the model. In summary, our contributions are:

- We propose low-light enhancement fast lane detection (LLFLD), a lane detection system that combines a low-light image enhancement network (ALLE) with a lane detection network. Our approach significantly enhances the performance of the network in low-light environments while maintaining an ultra-fast detection speed.
- We propose a symmetric feature flipping module (SFFM), which refines the low-level features and gains more precise lane localization.
- We propose a channel fusion self-attention mechanism (CFSAT) in the auxiliary segmentation module, which captures and utilizes more global context information.
- We propose a novel structural loss function that leverages the inherent geometric constraints of lanes to optimize the detection results.

## 2. Related Work

### 2.1. Traditional Methods

Autonomous driving heavily relies on the accurate detection of lanes, which serves as a fundamental task in achieving self-driving capabilities. This task involves identifying the lanes on the road and providing relevant information such as lane ID, direction, curvature, width, length, and visualization. Over the years, a plethora of computer vision technologies have been developed to achieve robust lane detection without making assumptions about the number of lanes present, involving the location and tracking of lane boundaries in road scenes. Traditional approaches typically utilize hand-crafted features, such as edge detection, the Hough transform, and color filtering [6,7,8]. However, these techniques are known to be susceptible to various environmental factors, including illumination changes, occlusions, and complex road layouts, rendering them less effective in challenging driving scenarios.

### 2.2. Segmentation Methods

Semantic segmentation represents an active research area in computer vision, with the primary aim of assigning a semantic label to each pixel in an image. Two primary categories of methods can be identified, namely region-based and end-to-end approaches. Region-based methods often leverage region proposal networks (RPNs) [9] or sliding windows to extract regions of interest (ROIs) [10] from the input image. The segmented network is then applied to each ROI to generate precise masks. VPGNet [11] exemplifies a region-based approach, which generates ROIs for lanes and other road markings using RPNs and applies a multi-task network for pixel classification within ROIs. End-to-end methods, on the other hand, directly apply a segmentation network to the entire input image without using any ROIs, which are more computationally efficient. In the context of end-to-end methods, the SCNN [3] approach employs a specialized convolution operation in its segmentation module, enabling the effective aggregation of features from different dimensions. This is achieved by processing sliced features and incrementally adding them together to effectively capture multi-scale contextual information. However, the method can be computationally expensive. To deal with the challenge of real-time applications, researchers have proposed lightweight semantic segmentation methods, such as self-attention distillation (SAD) [4]. To enable effective knowledge transfer between high level semantic information and low-level location feature attentions, SAD utilizes an attention distillation module that treats the former as a teacher and the latter as a student. LaneNet [12] employs an instance segmentation pipeline to handle a varying number of lines; however, the generation of line instances necessitates post-inference clustering.

### 2.3. Anchor-Based Methods

The anchor-based approach is a widely used technique for lane detection, where a set of predefined anchors is employed to localize the lane markings. Among different variants of this approach, the linear anchor-based method utilizes linear anchors as reference points for accurate lane regression. The pioneering work of Line CNN [13] introduced the use of line anchors in lane detection; later, Feng et al. [14] proposed a Bezier curve-based regression model to address the challenge of a large number of polynomial regression parameters. The Bezier curve model is not only computationally efficient but also highly stable. Lane ATT [15] proposed a novel attention mechanism based on anchor points, which aggregates global information and shows outstanding performance accompanied by high efficiency. Some recent methods employ classification based on row selecting [5,16] as a means of custom down-sampling segmentation in units of one pixel, but still require post-processing. Another noteworthy anchor-based method is UFLD [5], which frames the lane detection problem as a row anchor presetting and row selection task that leverages continuity of lane lines for improving detection speed. It exhibits ultra-fast detection speed, albeit with an accuracy that leaves room for further improvement.

### 2.4. Low Light Image Enhancement

The field of low-light image enhancement has achieved a relatively high level of development [17,18,19]. Histogram equalization and dehazing-based methods are the two primary techniques employed in traditional image enhancement methods. Histogram equalization is a classical method that enhances images by stretching their histogram distribution to achieve uniform brightness across the entire range. Zhang et al. [20] propose the utilization of various image processing techniques, including histogram equalization, as a data normalization method for machine learning. To evaluate the effectiveness of these techniques, the paper compares their performance to that of z-score normalization on a face-based authentication algorithm using SVM and random forest classifiers. FEBD [21] for multiple histograms modification presents a novel fast expansion-bins-determination method for multiple-histogram-modification-based reversible data hiding (RDH), which is a powerful technique that enables the embedding of data into images while maintaining the original information content. Specifically, the proposed method enhances the RDH approach by introducing an efficient algorithm capable of determining optimal expansion bins quickly and accurately, with only a negligible performance loss compared to the original method. Some approaches adopt the dehazing-based [22], which presents a new single image dehazing algorithm based on the dark channel prior. This method features an improved atmospheric light estimation method and a low-complexity morphological reconstruction technique to generate high-quality dehazed images with significantly reduced computational complexity compared to previous approaches. To solve the scattering of sunlight in the atmosphere, Ref. [23] proposes an image enhancement approach based on inverting low light images and applying image dehazing with an atmospheric light scattering model. Unlike traditional methods that rely on hand-crafted features, deep learning methods leverage the power of neural networks to learn a pixel mapping function to transform low-light images to high-quality outputs. Recently, Ref. [24] raised a new dataset of low-light images and videos, along with an online platform that comprises various popular methods for evaluation and comparison. Differing from the methods which need the reference of Retinex theory and labels [25], Zero-DCE [26] is a no labels approach that considers image brightness enhancement mapping in supervising by several no references luminosity and color loss function. Accordingly, the algorithm exhibits enhanced robustness, a wider range for adjusting the dynamic range of images, and reduced computational costs.

## 3. Methodology

### 3.1. Overall Pipeline

**Figure 1:**
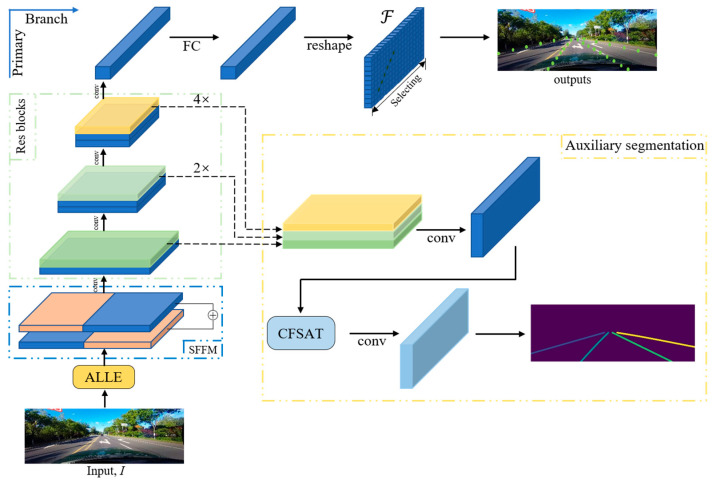
Overview of the proposed LLFLD. The pipeline consists of a primary classification detection branch and an auxiliary segmentation branch. Notably, the auxiliary segmentation module is exclusively activated during the training phase.

LLFLD is a lightweight lane detection model that integrates low light image enhancement techniques. Our approach achieves ultra-low computational costs by transforming the segmentation task into the selection and classification task of predetermined line anchors. An overview of the method is shown in Figure 1. Our framework comprises two parts: a primary classification detection branch and an auxiliary segmentation branch, designed to process RGB images $I\in {\mathbb{R}}^{3\times H\times W}$ captured from a forward-facing vehicle camera. The outputs of the system are selected lane boundary points corresponding to row anchors. To generate these outputs, the input images undergo automatic low-light enhancement before being fed to the classification detection branch, which includes a backbone ResNet stage. The low-level feature maps after convolution still retain strong location information, so prior to entering the backbone ResNet stage, we flip the feature maps symmetrically using the symmetric feature flipping module (SFFM) to enhance the visual information that is often lost due to occlusion and blur, thereby more effectively locating the lanes. Then, we obtain F$\in {\mathbb{R}}^{C_{lane}\times H_{slice}\times W_{slice}}$ by passing the feature maps output by the backbone through a fully connected layer and a reshape layer, where $C_{lane}$, $H_{slice}$, $W_{slice}$ are, respectively, the number of lanes in each image, and the numbers of row demarcation and column demarcation. Finally, we predict the final output channels using F by transforming the segmentation problem into an anchor-based selection classification problem, which improves the detection efficiency significantly.

The auxiliary segmentation branch performs feature map upsampling from different stages of the Resnet network at varying scales, and concatenates and convolves them, similar to the structure of FPN [27] that fuses high-level features on the top and low-level features on the bottom. We update the feature map after hierarchical fusion with global information through the channel fusion self-attention module (CFSAT) and generate segmentation results to assist parameter learning in the backbone classification branch. It is noteworthy that the auxiliary segmentation branch is exclusively active during training but deactivated during the test and detection phases. In this way, this approach not only facilitates parameter learning in the backbone branch but also preserves fast detection speed.

### 3.2. Automatic Low-Light Scene Enhancement (ALLE)

**Figure 2:**
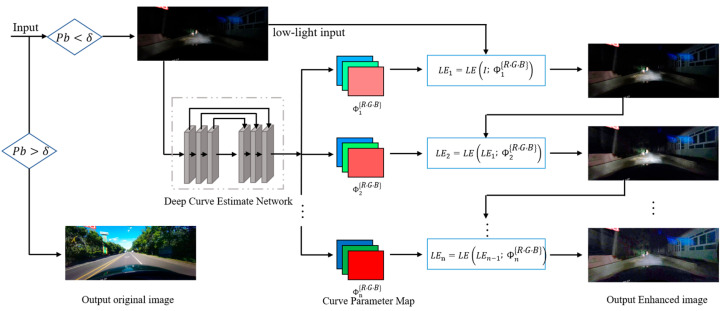
The framework of automatic low-light scene enhancement (ALLE). A Zero-DCE network is designed to inference a mapping $\Phi_{n}$ that corresponds to the optimal light enhancement tactics. The network enhances an input dark image iteratively, with n representing the number of iterations that regulate the extent of image enhancement. For normal exposure images, we recognize the original images as the outputs.

In practical applications, low brightness conditions can significantly impede visual perception, particularly in situations such as nighttime driving. The traditional method of improving image brightness often introduces more noise and lacks the necessary dynamic range and robustness. As a result, although it increases the visibility of the target lane lines, it also brings unwanted information that interferes with the detection results. To account for the complex and variable driving conditions, we recognize that not every image requires brightness enhancement. Additionally, applying the same enhancements to both normal and high brightness images can result in overexposure, which is also detrimental to the lane detection results. To overcome these challenges, our objective is to automatically enhance low-light images to achieve superior visual effects, with a specific emphasis on the detection performance of the enhancement model under low-light conditions. In this paper, we propose the automatic low-light scene enhancement (ALLE) module, which employs a Zero-DCE network to estimate the best mapping from low light image to normal exposure image, as depicted in Figure 2. We perform adaptive enhancement according to the brightness of different images, that is, enhance low-brightness images only, and maintain the same normal brightness and high-brightness images, rather than uniform or random preprocessing of all images. Initially, we calculate the root mean square (RMS) of RGB values of the input image $I\in {\mathbb{R}}^{3\times H\times W}$, which enables us to determine the perceived brightness Pb [28] of the image I in the RGB space, as represented by the following equation:

(1) $$Pb=f\left(R,G,B\right)=0.241R^{2}+0.691G^{2}+0.068B^{2}$$

where R, G, and B denote to the RMS-normalized RGB channel values of the input image I. Our study reveals that values of perceived brightness below 60, calculated using Equation (1), noticeably affect human visual perception. On the contrast, for values above 80, the perceived image brightness is considered normal. Thus, we apply deep enhancement processing to those images whose perceived brightness falls below the threshold δ, which has been set to 70.

To accomplish the task of low-light image enhancement, we leverage the Zero-DCE network, which formulates the problem as a non-reference multi-level recursive curve estimation task. The network adopts a lightweight encoder-decoder architecture for feature extraction and image enhancement generation. This approach enables end-to-end enhancement of images under low-light conditions. Furthermore, the perceived brightness of different images varies greatly, which hinders the training of a single network. Minimizing this variation as much as possible facilitates network optimization. Specifically, our objective is to train a mapping function $\Phi_{n}$, capable of transforming low-light images into normally exposed ones, as denoted by the following mathematical expression:

(2) $$LE_{n}\left(x\right)=LE\left(LE_{n-1};\Phi_{n}^{\left\{R,G,B\right\}}\right)=LE_{n-1}\left(x\right)+\Phi_{n}LE_{n-1}\left(x\right)\left(1-LE_{n-1}\left(x\right)\right)$$

The equation governing automatic low-light image enhancement entails a parameter map Φ that is trainable and shares the same dimensions as the input image. Here, x refers to the RMS-normalized channel values of the input image, while *n* denotes the number of iterations that regulate the curvature of the curve. The enhanced version of the final input, denoted as $LE_{n}\left(x\right)$, is obtained using the previous version $LE_{n-1}\left(x\right)$. Hence, the expression for $LE_{1}\left(x\right)$ can be expressed as:

(3) $$LE_{1}\left(x\right)=LE\left(x;\Phi_{}^{\left\{R,G,B\right\}}\right)$$

In order to learn the most-fitting enhance mapping utilizing ALLE network without any reference, we introduce a suite of differentiable luminosity and color loss functions that enable us to assess the quality of enhanced images while supervisory information is not required at all. Our ALLE network is trained using the following three types of loss functions.

**Intensity consistency loss.** The loss function $L_{Ints}$ aims to foster intensity consistency of the images after enhancement. This is achieved by minimizing the difference value between the adjacent domains of the dark images and their corresponding enhanced domains, as represented by the following equation:

(4) $$L_{Ints}=\frac{1}{M}\sum_{i=1}^{M}\sum_{j\in \omega \left(i\right)}{\left(\left|Z_{i}-Z_{j}\right|-\left|Y_{i}-Y_{j}\right|\right)}^{2}$$

where M represents the number of divided domains, and $\omega \left(i\right)$ denotes a set that contains the five domains including the center domain i and the upper, down, left, and right neighboring domains. The average intensity values of the five domains in the enhanced image and dark image are denoted as Z and Y, respectively.

**Exposure constrain loss.** To prevent the problem of over exposure or under exposure occurring in the enhanced version, we incorporate an exposure constrain loss function $L_{exp}$ to regulate the exposure level:

(5) $$L_{exp}=\frac{1}{N}\sum_{k=1}^{N}\left|Z_{k}-E\right|$$

where N denotes the number of divided domains of size 8 × 8 which do not overlap each other, $Z_{k}$ is the average intensity value of the k−th divided domains in the enhanced version, and E represents the satisfactory exposure degree of an image, which is set to 0.55. The function encourages the network to produce enhanced low-light images with satisfactory exposure domains and penalizes over exposure or under exposure domains.

**Color channel loss.** The color channel loss function $L_{col}$ is formulated as follows:

(6) $$L_{col}=\sum_{\forall p,q\in \varepsilon }{\left(J^{p}-J^{q}\right)}^{2},\varepsilon =\left\{\left(R,G\right),\left(R,B\right),\left(G,B\right)\right\}$$

where $J^{p}$ and $J^{q}$ denote the average intensity value of the p−th and q−th channels in the enhanced image, respectively. $\left(p,q\right)$ represents a pair of channels included $\left(R,G\right)$, $\left(R,B\right)$, and $\left(G,B\right)$. The color constancy loss function encourages the DCE network to produce enhanced images that are color consistent by minimizing the difference between color channels.

**Total enhanced loss**. The total loss is formulated as follows:

(7) $$L_{total\_e}=L_{spa}+L_{exp}+w_{col}L_{col}$$

where $w_{col}$ is the weight of the losses.

The proposed low light enhancement network (ALLE) is only appropriate for dimly lit settings and cannot be applied in other harsh weather conditions that hinder visibility, for instance, fog and torrential rain. To clarify, the developed ALLE optimizes low-light images to normal-light conditions, and is not designed as a mechanism to remove any obstructions that might impede clear sight.

### 3.3. Symmetric Feature Flipping Module (SFFM)

The global structure of lanes in driving scenes is examined from a first-person perspective, where it is observed that lanes manifest symmetry. Specifically, in non-curved scenarios, the presence of the left lane line implies the existence of the corresponding lane on the right, indicating a symmetric relationship. This relationship allows the structure of the left lane to inform the description of the missing visual information of the right lane, and vice versa. To leverage this symmetry property, a lane structural loss function is introduced to preserve the global structure of lanes in the enhanced image. This approach effectively enhances the visibility of both the left and right lane lines, thereby preserving the overall structure of the lanes.

To effectively utilize this observation, we propose the symmetric feature flipping module (SFFM) in this paper, as illustrated in Figure 1. The SFFM comprises two distinct convolution and normalization layers that transform each feature map independently. The transformed feature maps are then combined and passed through a ReLU activation function. Specifically, the lower-level feature map contains abundant position information. To obtain a new feature map, we perform a symmetric flip of the lower-level feature map along its longitudinal axis. As shown in Figure 1, the blue and orange regions of the feature map are symmetrically reversed and then concatenated together. A significant proportion of the examples in both our training set and test set consist of either straight or nearly straight slow turns. Approximately 70% of these turns are symmetric or nearly symmetric, 28% are not completely symmetric, and a small percentage of 1.2% are sharp turns. It is important to note that the road lanes are not strictly axisymmetric, but this processing step is implemented to improve the attention scores of the blocked and lost lanes. Notably, we employ deformable convolution [29] to replace the standard convolution operation to address problems caused by camera angle changes, such as jitter and rotation. The proposed module enhances the low-level features and gains more precise lane localization.

### 3.4. Channel Fusion Self-Attention Mechanism (CFSAT)

**Figure 3:**
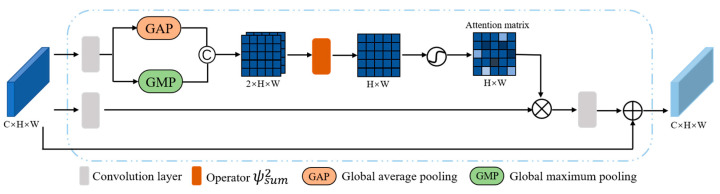
The framework of channel fusion self-attention mechanism (CFSAT).

Lane detection is a critical computer vision task that demands a balance between high level semantic information understanding and precise low level localization feature refining. To attain this balance, we propose a two-stage approach, wherein we first detect lanes using high-level semantic information and then refine the results based on low-level features. By leveraging the complementary nature of these stages, our method produces more accurate lane detections. In particular, we introduced the symmetric feature fusion module (SFFM) in the previous section to refine the low-level features. However, since a simple segmentation network may not be sufficient to gather adequate global context information, we developed the channel fusion self-attention mechanism (CFSAT) in the auxiliary segmentation branch, as demonstrated in Figure 3. This mechanism enhances the representation of advanced semantic features of lanes by incorporating more global context information.

The resulting updated feature map, denoted $M_{update}\in {\mathbb{R}}^{C\times H\times W}$, can be expressed by the following formula:

(8) $$M_{update}=M+Conv\left(SA\left(Conv\left(M\right)\right)\right)$$

In the equation above, the feature map before updating with channel fusion self-attention mechanism (CFSAT) is denoted as $M\in {\mathbb{R}}^{C\times H\times W}$. Conv represents a convolutional operation. To further enhance the feature map, an attention mechanism referred to as SA is employed to update the map with attention weights. The SA operation utilizes an attention matrix to weigh different features in the map, resulting in improved accuracy in lane detection. Mathematically, the SA operation is expressed as follows:

(9) $$SA\left(Conv\left(M\right)\right)=Conv\left(M\right)⊗softmax(\psi_{sum}^{2}\left(GA\left(Conv\left(M\right)©GM\left(Conv\left(M\right)\right)\right)\right)$$

where the symbol © represents the concatenation operation, while GA and GM denote global average pooling and global maximum pooling, respectively. The function $\psi_{sum}^{2}$ computes the square of the sum of corresponding pixels across channels. The choice of the $\psi_{sum}^{2}$ operator for channel fusion is based on the results of SAD. The SAD experiment revealed that using $\psi_{sum}^{2}$ operator for channel fusion yields maximum performance improvement.

### 3.5. A Novel Lane Structural Loss Function

**Figure 4:**
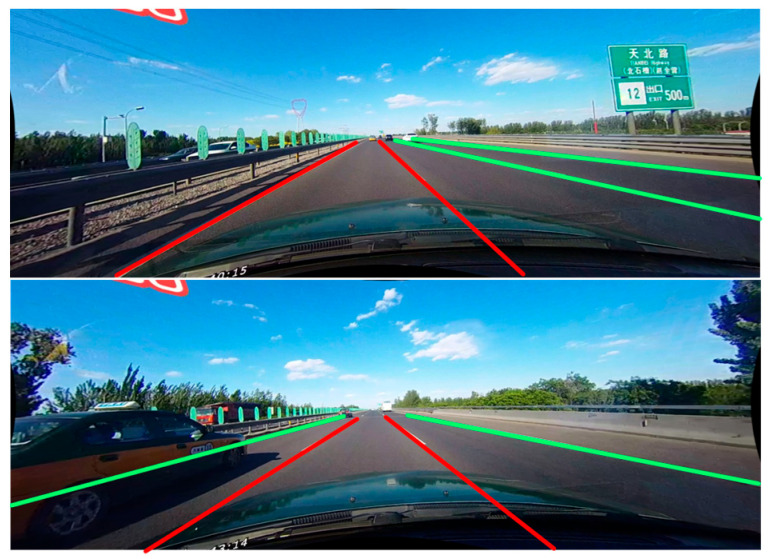
The illustration of design motivation of the structural loss function. The lane lines can be seen as a white strip in most conditions. From the perspective of driving, the absolute value of the slope of the lane lines on both sides of the driving lane (represented by the red lines) is always greater than the absolute value of the slope of the adjacent lane lines (represented by the green lines) due to the perspective principle.

Most detection models only utilize segmentation loss or classification loss to supervise detection results, disregarding the structure of lane lines. Nevertheless, we recognize that lane lines possess strong geometric prior information. Specifically, lane lines are typically thin, white strips with symmetric properties. Furthermore, due to the perspective principle, the absolute value of slope of the lane lines on both sides of the lane in which the vehicle is travelling is greater than that of adjacent lanes, as illustrated in Figure 4. In this paper, we introduce the probability $P_{i,j,k}$ to indicate the probability that a grid located at $\left(j,k\right)$ in the i−th lane lines belongs to the lane. To ensure differentiability of the function, we use the softmax function to obtain the probability of different locations:

(10) $$Probability_{i,j,:}=softmax\left(P_{i,j,1:w}\right)$$

where $Probability_{i,j,:}$ represents the probability at each location, $P_{i,j,1:w}$ is a w-dimension vector and 1: *w* indicates from column 1 to column *w*. It is worth noting that $P_{i,j,k}$ is a (w+1)−dimension vector, where the (w+1)−dimension indicates the probability of presence of a lane. The column localization $Pos_{i,j}$ of the i−th lane in the j−th row is transformed into a mathematical expectation, which is expressed as,

(11) $$Pos_{i,j}=\sum_{k=1}^{w}k\cdot Probability_{i,j,k}$$

where w represents the number of columns for partition. In practice, the localization points of lane lines between adjacent rows should be as close as possible. However, due to perspective relationships, there should be a geometric difference of α between the lane localization points of adjacent rows. To address this issue, we propose a novel structural loss function, denoted as $L_{struc}$, which can be expressed mathematically as follows:

(12) $$L_{struc}=\sum_{i=1}^{C}\sum_{j=1}^{h-1}\left|Pos_{i,j+1}-Pos_{i,j}\right|-\alpha \cdot M$$

where C denotes the number of lanes and h denotes the number of presetting row demarcation. Meanwhile, M is a binary mask with a value of 1 when $i=l_{1}$ or $l_{2}$, and 2 otherwise. Here, $l_{1}$ and $l_{2}$ correspond to the lanes with the maximum and second largest absolute values of slopes, respectively. Their calculation formulas are as follows:

(13) $$l_{1}=\underset{i}{\mathrm{argmax}}|\frac{Pos_{i,h}-Pos_{i,1}}{h-1}|$$

(14) $$l_{2}=\underset{i}{\mathrm{argsecmax}}|\frac{Pos_{i,h}-Pos_{i,1}}{h-1}|$$

To regulate the output results in the primary branch, we utilize the classification loss function denoted $L_{cls}$, which can be expressed as follows:

(15) $$L_{cls}=\sum_{i=1}^{C}\sum_{j=1}^{h}L_{CE}\left(P_{i,j,:},GT_{i,j,:}\right)$$

where $GT_{i,j,:}$ denotes the one-hot label of the correct locations, and $L_{CE}$ represents the cross-entropy loss function. In addition to $L_{cls}$, we also utilize cross-entropy as the auxiliary segmentation loss function, denoted as $L_{seg}$, in the auxiliary segmentation branch. Combining these loss functions, we obtain the overall loss of our method can be expressed as:

(16) $$L_{total}=L_{cls}+w_{seg}L_{seg}+w_{struc}L_{struc}$$

where $w_{seg}$ and $w_{struc}$ are the weights of segmentation loss function $L_{seg}$ and structural loss function $L_{struc}$, respectively.

## 4. Experiments

CULane [3] is one of the most comprehensive and challenging publicly available datasets for lane detection, which covers a wide range of scenarios and conditions. The dataset contains images with a resolution of 1640 × 590 pixels, and categorizes them into nine different classes according to the degree of difficulty and complexity. To evaluate the performance and robustness of our proposed method, we conducted extensive experiments on the CULane dataset, which comprises 133,235 images in total. The dataset is split into three subsets: a training set with 88,880 images, a validation set with 9675 images, and a test set with 34,680 images. In this section, we first introduce the evaluation metrics and some of the implementation details that are used in our experiments. Then, we report and analyze the experimental results on the CULane dataset and compare them with several state-of-the-art methods. Finally, we present an ablation study to investigate the effectiveness of different components and settings in our method.

### 4.1. Evalutaion Metrics

For the CULane dataset, the official metric used is the F1 score proposed in [3].

(17) $$F1=\frac{2\cdot Precision\cdot Recall}{Precision+Recall}$$

where $Precision=\frac{TP}{TP+FP}$ and $Recall=\frac{TP}{TP+FN}$. The CULane dataset is evaluated according to a specific protocol that involves representing the ground truth and predicted lane lines as 30-pixel-wide curves in the image space. This width of 30 pixels is used to define the lane lines of the dataset. The match was calculated for each whole line. A prediction line and a ground truth line are considered a match if their pixel IoU is over 0.5.

### 4.2. Implementation Details

In our implementation, we set the row demarcation for the CULane dataset according to the image height, which is 590 pixels. The row demarcation is a set of horizontal lines that divide the image into several regions for lane detection. We set the row demarcation to start from 0 pixels and end at 530 pixels with an interval of 10 pixels, by considering that the lane lines consistently appear at the bottom of the image (0 pixels high), but are not visible in the top portion of the image (around 530 pixels high), which typically features the sky, mountains, or distant objects in the driver’s view. The interval represents the number of divided rows, and each grid cell provides an anchor to be selected for lane prediction. To balance model efficiency and accuracy, we opted for a relatively modest interval and grid cell. Specifically, we set the interval to 10 pixels and the number of grid cells per row to 155 for this dataset.

We resized the images to 288 × 800 following the method in [3] in the optimization process. With a cosine decay learning rate strategy [30], we trained our model using the SDG-momentum optimizer [31], and the momentum was set to 0.9. The learning rate was initialized to 4 × 10^−4^. We employed ResNet as our backbone network, specifically utilizing ResNet18 and ResNet34. For this architecture, the convolution kernel size of the backbone network is 3 × 3, and both the stride and padding were set to 1. The loss coefficients $w_{seg}$ and $w_{struc}$ in Equation (15) are both set to 0.8 to balance the segmentation loss and the structural loss. We use a batch size of 16, and the model is trained for 60 epochs on the CULane dataset. All training and testing are performed using PyTorch 1.9 [32], an Intel(R) Core(TM) i7-4790K and an NVIDIA GTX 1080Ti GPU with 11 GB memory.

## 5. Results

**Figure 5:**
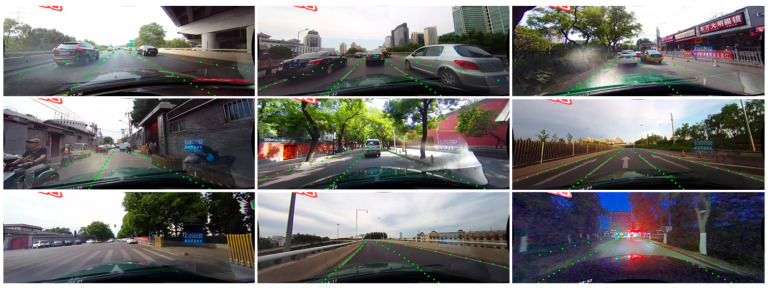
Qualitative results on the test set of CULane dataset. It shows nine road conditions in the CULane dataset, from left to right and top to bottom as normal, crowded, dazzle, no-line, shadow, arrow, cross, curve, and night.

We present the quantitative results of LLFLD on the CULane dataset in Table 1, where we compare our method with other advanced methods in terms of total F1 score and running speed. We also show some qualitative results of nine scenarios randomly selected from the test set of the CULane dataset in Figure 5, where we visualize the predicted lane lines by proposed methods. As can be seen from Table 1, our method achieves the best performance among all the compared methods on the CULane dataset, with an F1 score of 75.2% while running at 177 FPS. Compared to UFLD, which has the same research type as ours, our method has a significant advantage in accuracy while maintaining a comparable speed. For example, our lightweight model based on ResNet-18 achieves an F1 score of 72.6% while running at 330 FPS, while UFLD’s lightweight model based on ResNet-18 achieves an F1 score of 68.7% while running at 322 FPS on the same machine. Moreover, our method also surpasses the advanced method Lane ATT method, which uses an attention mechanism to enhance the feature representation for lane detection. For instance, our model based on ResNet-34 achieves an F1 score of 75.2% while running at 176.3 FPS, while Lane ATT’s model based on ResNet-34 achieves an F1 score of 74.1% while running at 140 FPS on the same machine. Furthermore, our method shows remarkable improvement in some challenging scenarios, such as night and shadow scenes. For example, in the “night” scene, our method achieves an F1 score of 66.0%, which is 3.4% higher than Lane ATT’s F1 score of 62.6%. Similarly, in the “shadow” scene, our method achieves an F1 score of 66.9%, which is 2.3% higher than Lane ATT’s F1 score of 64.6%. These results demonstrate that our framework and structural loss function can effectively handle the low-light and low-contrast situations and produce accurate and robust lane detection results, as our method can enhance images for low light scenes and utilize abundant global information and geometric information to tackle the no-visual-clue problems.

We show some visualizations of our proposed LLFLD method on the CULane dataset in Figure 5, where we display the lane lines predicted by our method on nine different road conditions. As can be seen from the figure, our method performs well under various scenarios and challenges, such as normal, crowded, dazzle and so on. The figure demonstrates that our method can accurately and robustly detect the lane lines under different illumination, occlusion, and curvature situations.

## 6. Ablation Study

This experiment aims to evaluate the impact of each major part of the proposed design on the lane detection performance. The four major parts are: automatic low-light scene enhancement (ALLE), symmetric feature flipping module (SFFM), channel fusion self-attention mechanism (CFSAT), and the proposed new structural loss function $L_{struc}$. To evaluate the performance of our proposed modules, we conducted a series of experiments under the same training settings but with different combinations of modules. The quantitative results of our modules, measured by the same metrics, are presented in Table 2.

**Figure 6:**
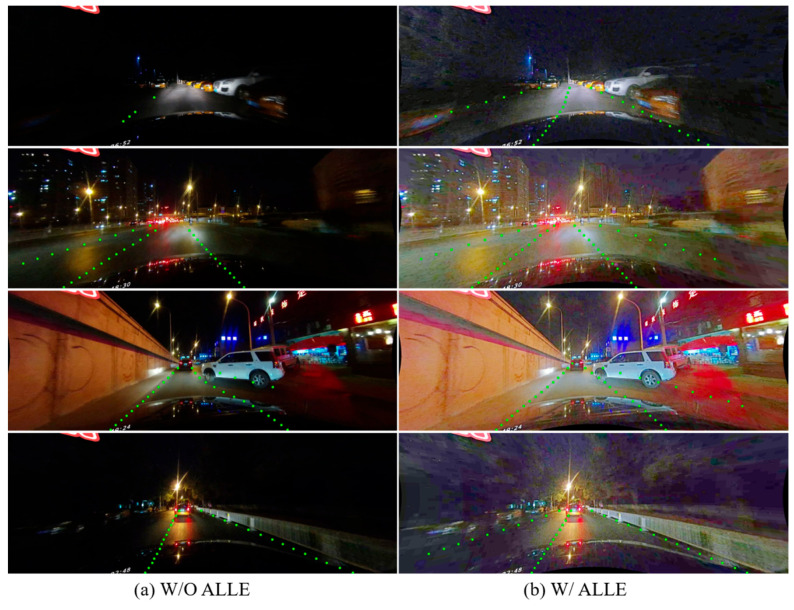
Qualitative comparison of detection results with and without automatic low-light scene enhancement (ALLE). The detection results of the night scenario in the same lane are shown. (**a**) shows the visualization of results without ALLE, while (**b**) shows the counterpart with ALLE.

To demonstrate the effectiveness of the ALLE module, which is a novel adaptive low-light enhancement module that can adjust the brightness and contrast of the input image according to the ambient illumination, we conducted a comparative experiment on the CULane dataset with night scenes. We compared the detection results before and after turning on the ALLE module on the CULane dataset in night scenes, as shown in Figure 6.

To assess the efficacy of the ALLE module in low-light scenarios, we utilized F1 values in shadow and night scenarios as our evaluation indicator. Table 3 illustrates the ablation experiments performed under two states—with ALLE activated and with ALLE deactivated. From Table 3, we can reach the conclusion that ALLE being activated achieves a significant improvement in performance compared to ALLE being deactivated, which provides evidence for the effectiveness of the proposed module in the low-light scenarios, such as shadow and night.

From Table 2, we can reach the conclusion that all proposed designs achieve a significant improvement in performance compared to the baseline, which provides evidence for the effectiveness of the proposed modules.

It can be observed that Figure 6 visually demonstrates that the detection results of the model with ALLE are better than those without ALLE in the same low-light scenes, which provides more intuitive evidence for the effectiveness of ALLE. Specifically, the ALLE module can help the model detect lanes accurately, even in low-light conditions when they may not be visible to the human eyes. This is a significant advantage of the proposed method, as it can improve the safety and reliability of lane detection in real-world scenarios with low-light conditions.

## 7. Conclusions

In this paper, we proposed LLFLD: a novel lane detection model specifically designed for enhancing low light scenes based on row anchor selection. We introduced the automatic low-light scene enhancement (ALLE), an adaptive low-light enhancement module that can adjust the brightness and contrast of the input image, according to the ambient illumination, to optimize the lane detection results in low-light scenarios. Our approach leverages feature flipping and channel self-attention mechanisms to effectively collect and utilize both low-level location information and high-level semantic information from the feature maps. Additionally, our new structural loss function leverages the geometric priori of the lane to optimize the detection results. Moreover, our design based on row selection ensures that the model is fast and lightweight, which is suitable for real-time applications. Experimental results on the popular CULane dataset demonstrate the favorable performance (measured by F1 score) of our proposed model. Furthermore, our method achieves fast inference speed and is lightweight. Specifically, the ResNet-34 version of our method can achieve 177 FPS while maintaining comparable performance at the same resolution. Overall, our proposed method is effective in enhancing low-light scenes for better lane detection results while maintaining fast inference speeds and low computational complexity.

## Figures and Tables

**Table 1:** Comparison of F1-measure and FPS on CULane testing set with IoU threshold = 0.5. For crossroads, only false positives are shown. The less, the better. ‘-’ means the result is not available. The best and second-best results across methods are in bold and underlined, respectively. All the images are resized to 288 × 800, and all the experiments were computed on a machine with an Intel(R) Core(TM) i7-4790K CPU and an RTX1080Ti GPU.

Method	Total	Normal	Crowded	Dazzle	Shadow	No line	Arrow	Curve	Cross	Night	FPS	MACs(G)
Res50-Seg [33]	66.70	87.43	64.10	54.12	60.70	38.10	79.00	59.83	2505	60.60	-	-
LSTR(ResNet-18,2×) [34]	68.72	86.78	67.34	56.63	59.82	40.10	78.66	56.64	**1166**	59.92	-	-
FastDraw (ResNet-50) [35]	67.13	85.90	63.60	57.00	59.90	40.60	79.40	65.20	7013	57.80	90.3	-
SCNN [3]	71.60	90.60	69.7	58.50	66.90	43.40	84.10	64.40	1990	66.10	7.5	328.4
ENet-SAD [4]	70.8	90.10	68.80	60.20	65.90	41.60	84.00	65.70	1998	66.00	75	7.8
UFLD(ResNet-18) [5]	68.40	87.70	66.00	58.40	62.80	40.20	81.00	57.90	1743	62.10	322.5	-
UFLD(ResNet-34) [5]	72.30	90.70	70.20	59.50	69.30	44.40	85.70	**69.50**	2037	66.70	175.0	-
CurveLanes-NAS-M [36]	73.50	90.20	**70.50**	**65.90**	69.30	**48.80**	85.70	67.50	2359	68.20	-	35.7
**Res18-Ours**	71.30	89.20	67.20	58.50	63.30	42.50	82.80	58.00	1819	66.50	330	17.4
**Res34-Ours**	**75.20**	**91.00**	71.80	65.30	**70.20**	47.80	**86.20**	**69.50**	1913	**70.50**	177	33.2

**Table 2:** Performance comparison of proposed modules on CULane dataset with ResNet-34 backbone. The baseline indicates that none of the modules are added. The F1-measure is computed on the CULane testing set with an IoU threshold of 0.5.

Baseline	Low-Light Enhancement	Flipping Module	Attention Mechanism	Structural Loss	F1
√					72.1
	√				73.8 (+1.7)
	√	√			74.3 (+2.2)
	√	√	√		74.9 (+2.8)
	√	√	√	√	75.2 (+3.1)

**Table 3:** Quantitative comparison of detection results on “shadow” and “night” scenarios with and without automatic low-light scene enhancement (ALLE). The F1-measure is computed on the CULane testing set with an IoU threshold of 0.5.

W/O ALLE	W/ALLE	Shadow-F1	Night-F1
√		68.90	65.73
	√	70.20	70.50

## Data Availability

Publicly available datasets were analyzed in this study. This data can be found here: https://xingangpan.github.io/projects/CULane.html (accessed on 23 October 2022).
